# Economic-Oriented Stochastic Optimization in Advanced Process Control of Chemical Processes

**DOI:** 10.1100/2012/801602

**Published:** 2012-11-01

**Authors:** László Dobos, András Király, János Abonyi

**Affiliations:** Department of Process Engineering , University of Pannonia, Egyetem Street 10, 8200 Veszprém, Hungary

## Abstract

Finding the optimal operating region of chemical processes is an inevitable step toward improving economic performance. Usually the optimal operating region is situated close to process constraints related to product quality or process safety requirements. Higher profit can be realized only by assuring a relatively low frequency of violation of these constraints. A multilevel stochastic optimization framework is proposed to determine the optimal setpoint values of control loops with respect to predetermined risk levels, uncertainties, and costs of violation of process constraints. The proposed framework is realized as direct search-type optimization of Monte-Carlo simulation of the controlled process. The concept is illustrated throughout by a well-known benchmark problem related to the control of a linear dynamical system and the model predictive control of a more complex nonlinear polymerization process.

## 1. Introduction

Due to the dynamic and significant changes of the economic environment performance assessment of process control is highlighted area of chemical engineering [1]. The aim of this paper is to develop an optimization framework designed to determine optimal operating regimes of chemical processes by taking process constraints, desired maximum number (frequency) of constraint violations, and process uncertainties into consideration. 

Variance in the closed control loop caused by unmeasured disturbances and badly designed controllers might cause variations in the product quality. In case of increasing variance of process variables the probability and frequency of violation of quality requirements are increasing that might lead to the increase of the amount of less valuable offset products. Typical examples when reduced number of violations of the predetermined process constraints are acceptable can be found in the field of statistical process control (SPC [2]). In Statistical Process Control statistical tools are applied to monitor the performance of the production process and detect significant deviations that may later result in offset products.

In this paper a more sophisticated model-based approach is followed. Modern process analysis, monitoring, control, and optimization tools are mainly based on some kind of process model. It is obvious to utilize these process models also in the economic assessment and optimization. Usually the output of cost-benefit analysis is cost reduction or profit increment expressed by a cost function. These functions incorporate the costs of the operation, raw materials, current prices of products [3], and risks of malfunctions. In our economic-oriented optimization strategy the aim is to find steady state operation points (controller set points) where profit might be realized. This task is fulfilled at the supervisory control level [4].

The general approach for economic performance evaluation comprises the following steps: reduce the variance of the controlled variable and shift the set points (process mean) closer to the operation limits [5] without increasing the frequency of the violation. This operation is referred to as the improved control [6]. The variance reduction might mean to retune the existing controllers, or, in more radical cases, change the whole control strategy. The model-based predictive controllers (MPCs [7]) is highly applicable for variance-reduction purposes. Application of MPCs in the operative control level results in a multilayer optimization problem, since an MPC also minimizes its objective function. In this approach the upper layer is the supervisory control level which is responsible for economically optimal operation, and the lower layer is for variance reduction.

To handle uncertainty and effects of measurement noise in this paper a novel Monte-Carlo simulation-based approach is proposed. Monte-Carlo simulation is frequently applied in various areas [8]. This tool has also proven its efficiency in risk-related optimization of chemical processes; for example, it is applied in optimizing maintenance strategies of operating processes [9]. There is a common characteristic in these solutions: the stochastic nature of the studied system has to be modeled. In the applied methodology this simulation is related to the modeling of the unmeasured disturbances of the control loops. To handle this random effect, Monte-Carlo simulation is applied with the characterized noise. An economic cost function is calculated in every case to measure the economic efficiency of the process. Integrating this benefit analysis tool into the mesh adaptive direct search optimization algorithm—where the task is to find the most beneficial steady state operation point—resulted in the proposed economic-oriented optimization framework. In the proposed multilayer optimization framework, the application of gradient-based methodologies for maximizing the economic throughput is not possible, thanks to the stochastic characteristics caused by the closed-loop variance. That is why the utilization of direct search methods is necessary. Mesh Adaptive Direct Search (MADS) [10] class of algorithms is a relatively new set of direct search methods for nonlinear optimization; that is, these algorithms are capable of calculate the extremums of a nonsmooth functions, like our economic objective function. Since the steady state operation points are mainly determined by the variance of the controlled variables, incorporating this effect into the model is inevitable. The created optimization framework functions as an industrial Advanced Process Control system, [3, 11].

The paper is organized as follows: in Section 2 the economic cost function-based multilayer optimization framework is introduced. In Section 3 the applied methodology is explained in detail. In Section 4, the efficiency of the proposed methodology is illustrated throughout a linear benchmark control problem and a Model Predictive Controlled (MPC) highly non-linear technology. In both cases an economic performance measure has been formalized as a basis for optimizing the set point signal. As base case of the benchmark example the process is controlled with a PI controller. To reduce the closed-loop variance caused by unmeasured disturbance a linear MPC is installed to replace the PI controller. With the reduction of the variance the set point of the controller can be moved closer to the process constraints which yields higher economic performance. Such economic-oriented optimization is carried out at two different risk levels. As a second example a non-linear process controlled by a linear MPC is considered, since this combination is widely applied in chemical process industry. In this case study the process variance is caused by an unmeasured disturbance, model mismatch, and noise added to the controlled variable. In this example the effect unmeasured disturbance with different amplitude is examined in detail. These examples show the realistic benefits of the proposed methodology.

## 2. Economic Cost Function-Based Multilayer Optimization

**Figure 1:**
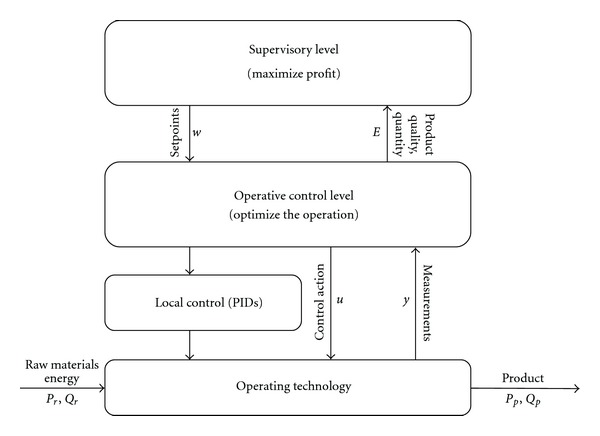
The layers of an economic optimization of an operating technology.

The proposed framework is rather similar to Advanced Process Control (APC) systems applied in the chemical process industry, [3]. The scheme of this multilayer optimization problem is depicted in Figure 1. The main aspects and tasks that have to be taken into consideration in the different optimization levels will be introduced in the following subsections.

### 2.1. The Supervisory Control Level

**Figure 2:**
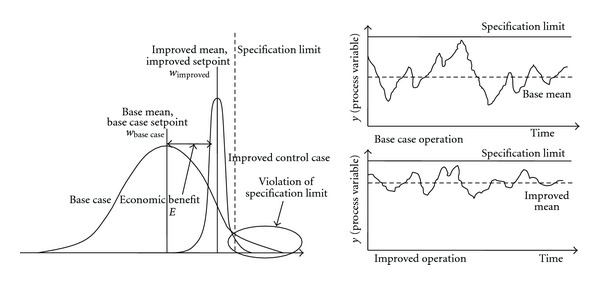
Approach to economic benefit estimation with variance reduction.

The main task in the supervisor level is to maximize the economic throughput with varying the steady state set point signal. In general the economically optimal set point is close to the operation limits of process. That is why the reduction of the closed-loop variance is necessary. Thanks to process variance—caused by disturbances, noise, and so forth—there is a risk of process constraint violation which has to be taken when the new set point is determined. The essence of this economic optimization approach is depicted in Figure 2.

The aim of the economic-oriented process optimization framework can be formulated as maximizing a cost function. Such cost function mostly includes the cost of the actual operation, raw materials, and the value of the product as the following:

(1) $$\begin{array}{c} \underset{w}{\max}E=\sum_{i=1}^{N}P_{i}\cdot Q_{i}-\sum_{r=1}^{M}P_{r}\cdot Q_{r}, \end{array}$$

where *P*_*p*_, *P*_*r*_ and *Q*_*p*_, *Q*_*r*_ are the prices and the quantities of the products and raw materials, respectively. In the optimization problem **w** = [*w*_1_,…,*w*_*p*_]^*T*^ represents the setpoints of local controllers of the operative control level; *p* is the number of the controlled variables, denoted with *y*_*i*_. The task of the operative control level can be summarized as *y*_*i*_ should be as close to *w*_*i*_ as possible.

Continuous economic improvement of process control is about to find the setpoint values of control loops with the possible highest economic performance. In steady state operation the value of (1) is often increasing by shifting the steady state operation point closer to the constraints of the process. To reach this goal, the reduction in variance of the key process variables is necessary with, for example, retuning the controller or even redesigning the existing control strategy. As Figure 2 illustrates, when the variance of the key process variables is reduced extra profit can be realized as the difference of the economic potential in the old and revised steady state operation points.

The cost function must be optimized with respect to the process constraints to ensure the required process safety and product quality. Constraints defined on the process variables can be expressed as follows:

(2) yi,min⁡≤yi,≤yi,max⁡, i=1,…,p,uj,min⁡≤uj,≤uj,max⁡, j=1,…,m,

where *y*_*i*,min⁡_ and *y*_*i*,max⁡_ are the lower and upper bounds of output variables, *u*_*j*,min⁡_ and *u*_*j*,max⁡_ are the lower and upper bounds of input variables, and *p* and *m* are the number of output and input variables, respectively. Thanks to uncertainties, such as process variation and disturbance, the probability of violating the predetermined process constraints is increasing by getting closer to them. A reasonable approach to handle the uncertainties in the constraints is to cast the the problem in term of the probability of constraint violation, which is the approach to be implemented in this paper.

The probability constraints can be expressed as

(3) $$\begin{array}{c} \text{Pr}\left\{y_{i,\min}\leq y_{i}\leq y_{i,\max}\;\;i=1,\ldots ,p\right\}\geq 1-\alpha , \end{array}$$

or

(4) $$\begin{array}{c} \text{Pr}\left\{y_{i,\min}\leq y_{i}\leq y_{i,\max}\right\}\geq 1-\alpha_{i}\;i=1,\ldots ,p, \end{array}$$

where Pr{·} is the operator of probability and *α* is the specified probabilistic violation level (demonstrated in Figure 2). The formulation of probability constraints means that satisfying process constraint of *y*_*i*_ is not required by 100% probability but a certain confidence level, 1 − *α*. Inequality (3) represents a so-called Joint Probabilistic Constraint (JPC) problem, which means that all process variables must be kept in the defined operation regime with maximum probability of violation of *α*. Inequality (4) is called is Individual Probabilistic Constraint problem (IPC) ([6]), where each process variable has a specified confidence level, 1 − *α*_*i*_, to be satisfied. In this paper the second approach has been adopted.

The final goal in multilayer optimization is to maximize the accessible profit, determined by the cost function mentioned before in (1), by finding the optimal steady state operation point with respect to the process constraints, (2). Thanks to process variation a reasonable risk level has to be taken by defining the probability of process constrain violation (formalized as *α*). 1 − *α* means a confidence level, which is a non-linear constraint in the economic optimization. This optimization problem represents the supervisory control layer.

### 2.2. The Operative Control Level

In optimization and control of complex production processes, the role of Model Predictive Controllers (MPCs) is increasing. The more and more widespread application is reasonable, thanks to the good variance reduction ability.

MPC is a model-based control algorithm where models are used to predict the behavior of process outputs of a dynamical system with respect to changes in the process inputs. The MPC uses the models and current plant measurements to calculate future moves in the manipulated variables, which will result in operation that honors all input and output variables' constraints (see (2)).

**Figure 3:**
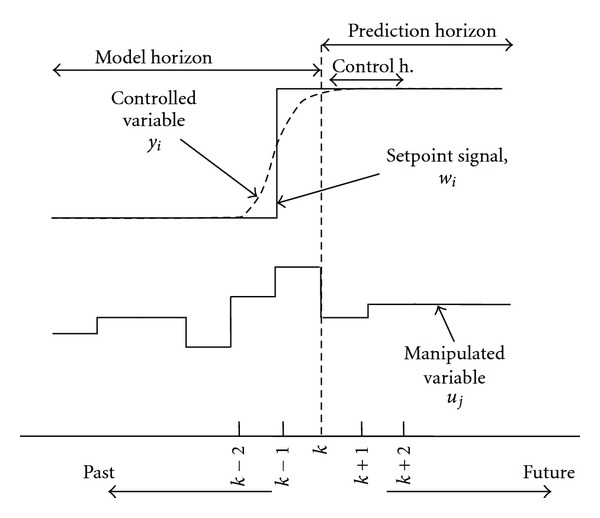
Illustration of essence of MPC in control of *y*_*i*_ by manipulating *u*_*j*_.

Predictive control uses the receding horizon principle. This means that after the computation of the optimal control sequence, only the first control action will be implemented; subsequently, the horizon shifted one sample and the optimization is restarted with new information about the measurements. That is the reason why the MPCs do not optimize the operation on the time horizon of the whole steady state operation, but consider just the horizon, implemented in the controller, and solve the optimization problem iteratively. With the help of Figure 3 the essence of the model predictive control is easily understandable.

Utilizing this control strategy in the operative control level the previously proposed multilayer optimization framework can be resulted (depicted in Figure 1), thanks to the control rule of MPCs, expressed as:

(5) $$\begin{array}{c} \underset{\Delta u_{\left(k+j\right)}}{\min}\sum_{i=1}^{p}\;\sum_{j=1}^{H_{p}}{\left(w_{i,k+j}-y_{i,k+j}\right)}^{2}+\sum_{i=1}^{m}\lambda_{i}\sum_{j=1}^{H_{c}}\Delta u_{i,k+j-1}^{2}, \end{array}$$

where *p* and *m* are the numbers of the controlled variables and manipulated variables, respectively. In MPC control strategy the different number of controlled and manipulated variables is acceptable, since the interconnection between the different manipulated and controlled is considered in the process model, which is applied in the MPC. The tuning parameters of the controller are as follows: *H*_*p*_ and *H*_*c*_ are the length of prediction and control horizo, *λ* is the a factor for punishing the change of the control signal. Δ**u** is the variation of the manipulated variable at a given time, which is calculated during the optimization method on *H*_*c*_ control horizon.

MPC formulates an objective function which is used to find the optimal input sequence to eliminate the difference of the controlled variable and the set point in the prediction horizon. Since this objective function, (5), is designed to ensure smooth and stable operation it does not directly reflect the economic performance of the technology (formalized in (1)). Additionally this cost function does not count to the risk of violating the process constraints caused by unmeasured disturbances which appears as closed-loop variance (see (3)-(4)).

Constraint violations have also to be taken into account during economic performance optimization. This is the reason why this paper suggests the application of Monte Carlo simulation of the augmented process model and the model of the operative control level (see Figure 1). The result of the Monte Carlo simulation is an aggregated economic performance (e.g., mean of the economic performance of the individual runs). Due to the stochastic nature of the optimized system the gradient of this aggregated economic cost function is difficult to calculate. Hence the optimization algorithm should be gradient free-yet computationally very effective. To meet this requirement the application of the advanced Mesh Adaptive Direct Search methodology is proposed. In the following section the multilayer optimization framework and its two main building blocks—Monte Carlo simulation and Mesh Adaptive Direct Search methodology—are going to be introduced in detail.

## 3. Stochastic Modeling Economic Benefit Maximization with Direct Search Methodology

Taking process variance into account the previously proposed economic-oriented objective function, (1), becomes a stochastic characteristic. To handle uncertainties Monte Carlo simulation is applied. The Monte Carlo method is applied frequently in solution of stochastic optimization problems, for example, in stochastic linear programming [12, 13]. Kjellstrom [14] was the first to use Monte Carlo estimators for the iterative improvement of convergence behavior in nonlinear stochastic optimization.

Due to the stochastic characteristics caused by the closed-loop variance the application of gradient-based methodologies for maximizing the economic throughput is not efficient. Integrating the simulation-based economic performance assessment methodology into a direct search optimization algorithm an effective optimization framework is obtained. Mesh Adaptive Direct Search (MADS) [10] class of algorithms is a relatively new set of direct search methods for nonlinear optimization; that is, these algorithms are capable of calculating the extremums a nonsmooth objective functions, like our economic objective function.

Our methodology is stated as follows:

- economic performance assessment of the considered steady state operation point. It means applying a set point (**w**) and calculating the value of the economic cost function, (1), with respect to the process constraints, (2), and the value of the probability of constraint violation (see (3)-(4)). Because of considering the process variance as random phenomena, Monte Carlo simulation with multiple runs of augmented process simulator is applied to aggregate the effect of the random variances in a final economic cost function;
- integrate the economic performance evaluation tool into the MADS optimization algorithm to find the economically optimal steady state operation point. The previously applied economic cost function, (2), has to be maximized with respect to the proposed constraints with varying setpoint signal (**w**). This algorithm can handle constraint limits of process variables, the certain confidence levels to violate these limits.

Using the methodology discussed above the optimization process is capable to isolate and handle all the disturbances technology has, whose nature is constant in time; thus it can be characterized statistically. These uncertainties are time homogeneous and static-time disturbances, like measurement noise or model error. In the following section the application way of Monte Carlo simulation and MADS optimization algorithm is introduced briefly.

### 3.1. Monte Carlo Simulation

 Monte Carlo Simulation (*MCS*) methods are highly applied in the mathematical modeling problems where some kind of stochastic phenomena must be handled. In the proposed multilayer optimization framework process variance caused by unmeasured disturbances is considered. The Monte Carlo simulation consists of the following steps.

- Define the domain of possible inputs.
- Generate inputs from this domain randomly using a specified probability distribution.
- Execute deterministic computation using the inputs.
- Aggregate the results of the computations into the final result.

In engineering practice normal distribution is considered as an adequate assumption for characterizing uncertainties. At the modeling of the considered process the following steps are followed: at first the mathematical model of the process is created. Then noise and unmeasured disturbances of the control loops are characterized and random signals related to the real process variance are added to the corresponding input and output variables. The value of the economic objective function, (1), is calculated by aggregating the results of the individual Monte Carlo runs into a statistical economic performance. Since complex production processes are mostly characterized by non-linear process models the economic assessment and optimization needs an optimization algorithm which is able to handle the non-linear cost functions and constraints, (2), (3), and (4).

### 3.2. The Mesh Adaptive Direct Search Methodology

Since the calculation of the gradient of the economic objective function with respect to the steady state operation points is highly computational demanding and due to the Monte Carlo simulation the economic cost function is nonsmooth the application of gradient free optimization method is needed. Mesh Adaptive Direct Search (MADS) [10] is a relatively new set of direct search methods for nonlinear optimization. This algorithm is capable of minimizing a nonsmooth function, like our economic cost function (1) under the proposed constraints in (2) and (4). According to [10, 15], MADS can be interpreted as a generalization of Generalized Pattern Search (GPS) [16] algorithms, with the restriction to finitely many pool direction removed.

MADS is an iterative algorithm, where at each iteration a finite number of test points are generated. At the beginning of an iteration, the infeasible test points are filtered (discarded); that is, infinite objective value is assigned to it (*f*(*x*) = +*∞*). Thereafter the feasible test points are evaluated by the objective function and compared with the current best objective function value found so far. Each of these test points lies on the current mesh, which is constructed from a finite set of *n*_*D*_ directions *D* ∈ ℝ^*n*^ and scaled by the mesh size parameter Δ_*k*_^*m*^ ∈ ℝ^*n*^. If we find a point with lower objective value than the current best one, this test point is a so-called *improved mesh point* and the iteration is a *successful iteration*.

**Figure 4:**
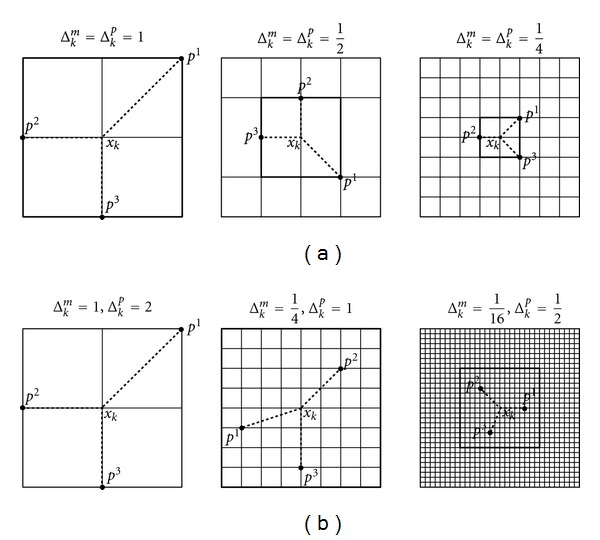
Example of GPS frames (a) and MADS frames (b) *P*_*k*_ = {*x*_*k*_ + Δ_*k*_^*m*^ *d* : *d* ∈ *D*_*k*_} = {*p* ^1^, *p* ^2^, *p* ^3^} for different values of Δ_*k*_^*m*^ = Δ_*k*_^*p*^. In all six figures, the mesh *M*_*k*_ is the intersection of all lines.

Each iteration consist of two steps, the so-called SEARCH step and POLL step. SEARCH step can return any point of the underlying mesh; it is trying to find an unfiltered point. If it fails to generate an improved mesh point, then the second step, the POLL is invoked. POLL step consists of a local exploration around the current best solution, and the test points are generated in some directions scaled by the mesh size parameter. MADSs are novel in the number of usable directions, since In GPS, POLL directions belong to a finite set, while POLL direction in MADS belongs to a much larger set; in fact if the iteration number *k* goes to infinity, the union of the normalized POLL directions over all *k* becomes dense in the unit sphere. According to [10], this algorithmic construction allows stronger convergence. Another important difference between MADS and GPS is the so-called *poll size parameter*, Δ_*k*_^*p*^. This parameter determines the size of the frame where the POLL step can operate. In case of GPS, mesh size and poll size are equal (Δ_*k*_^*m*^ = Δ_*k*_^*p*^), while in MADS these two parameters can differ. This difference is depicted in Figure 4. Additional pieces of information like convergence analysis or practical implementations can be found in [15]. 

In the economic-oriented multilayer optimization framework (see Figure 1) MADS is applied in the supervisory level to maximize the economic performance formalized as (1). The optimization problem is solved with respect to the the process constraints, (2), and the value of the probability of constraint violation, see (3)-(4), with varying setpoint signal (**w**). Since MADS needs a reduced number of runs of the augmented process simulator, the optimal value of the setpoint signals can be quickly obtained. The low number of iteration during optimization is necessary, since Monte Carlo simulation of the operative control level (augmented process simulator) is applied, which is highly computation demanding process.

In the following section the effective application of the proposed framework is going to be examined throughout the case studies of a benchmark, linear process, and an MPC controlled highly non-linear technology.

## 4. Application Examples

 In this section, two application examples are presented to demonstrate the applicability of the proposed framework for enhancing the economic benefit of the operating technologies. The calculations for both examples are based on closed-loop data, generated using Matlab-Simulink. The uncertainties are presented in the examples as noise superimposed to inputs and outputs.

### 4.1. A SISO Process

**Figure 5:**
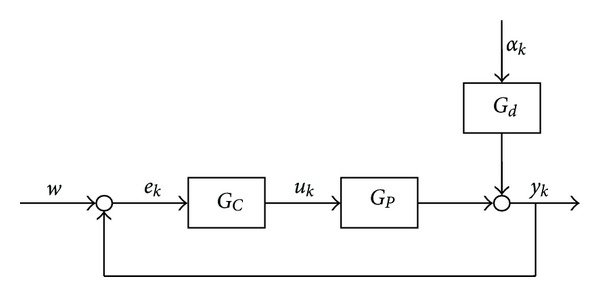
Block diagram of the SISO closed-loop system.

 Consider a SISO process, characterized by *G*_*p*_ shown in Figure 5 subject to disturbance dynamics *G*_*d*_ described by

(6) yk=Gpuk+Gdαk=0.6299z−11−0.8899z−1uk−2 +1−0.8z−11−0.8899z−1αk k=1…q,

where *α*_*k*_ is a normally distributed white noise sequence of mean 0 and variance 1. *q* signs the last time step of the considered simulation. The objective in the supervisory control level is to maximize the output ($\bar{y}$—mean of the output on the considered time horizon) with respect to the process constraints. The optimization problem can be formalized as

(7) $$\begin{array}{c} \;\underset{w}{\max}2\bar{y} \end{array}$$

subject to

(8) −10≤yk≤10−5≤uk≤5k=1…q.

**Figure 6:**
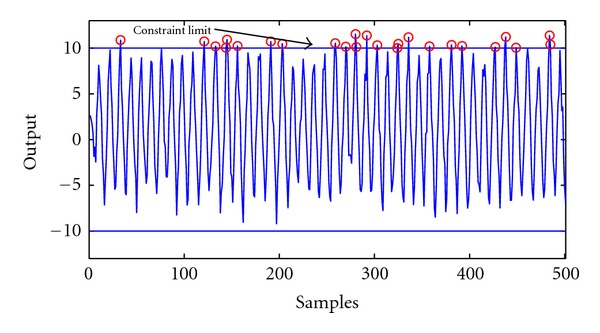
Base case operation with probability constraint level of 95%.

As base case a PI controller is designed. The controller parameters are *K*_*c*_ = 1.926*T*_*I*_ = 0.6. As previously shown, specifying the probability of not violating the constraint defined on the output variable defines a non-linear constraint for the optimization problem. During the presented studies this confidence level is assigned as 95% and 90%. In the literature [6] the same SISO process is utilized with the same probabilities. The means of the output are $\bar{y}=1.49$ and $\bar{y}=2.72$ confidence level of 95% and 90%, respectively. The output data in 95% confidence level is depicted in Figure 6.

**Figure 7:**
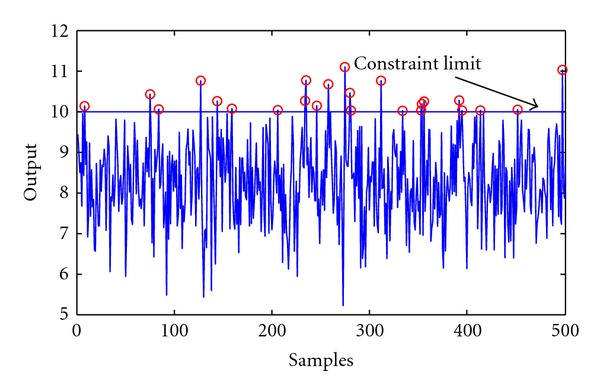
Improved case, optimal operation with probability constraint level of 95%.

**Figure 8:**
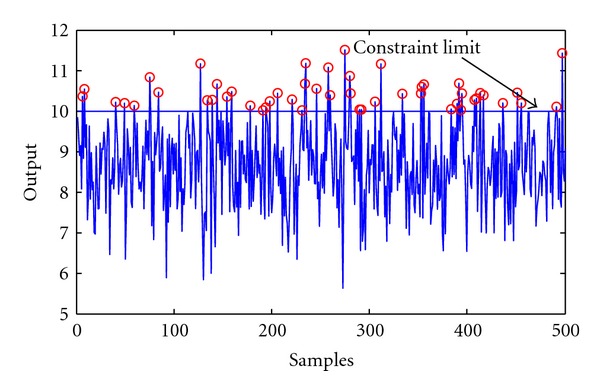
Improved case, optimal operation with probability constraint level of 90%.

Since MPC is highly applicable for variance reduction purposes the PI controller has been replaced with a linear Dynamic Matrix Controller (DMC) [17]. DMC applies the linear convolution model of the process for predicting the effects of the considered manipulated variable sequence. With the application of DMC lower variance (${\bar{\sigma }}_{PI}=5.3$ in contrast to ${\bar{\sigma }}_{\text{DMC}}=1.05$) and higher economic benefit might be expected. The control rule of the DMC has been proposed in (5). The tuning parameters of the applied DMC are *H*_*m*_ = 50, *H*_*p*_ = 20, and *H*_*c*_ = 10. The value of *λ* is chosen as 1000. By applying the previously proposed multilayer optimization framework significant improvement could be experienced in the economic performance (the number of Monte Carlo iterations was set to 100). The means of the output are $\bar{y}=8.28$ and $\bar{y}=8.68$ at confidence level of 95% and 90% respectively. The outputs in the improved operation are depicted in Figures 7 and 8. It can be clearly stated when the confidence level decreases the frequency of constraint violation increases. 

The number of individual economic performance evaluations in the Monte Carlo-simulation has been set to 100. There has been an attempt to apply quadratic programming as optimization algorithm (utilizing Matlab, Optimization Toolbox), but the computational demand was extremely high, almost one hour even in this simple example. By applying MADS, the computation demand has been significantly decreased into 5 minutes. In both cases the initial setpoint for the optimizer was set equal to the upper constraint of the output variable, *w*_0_ = 10.

**Figure 9:**
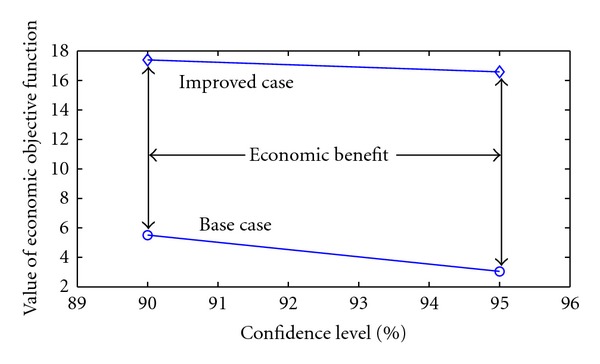
The economic performance in the base and improved case at different confidence levels.

In Figure 9 achievable economic benefit is depicted. Thanks to the replacement of the PI controller with the DMC the variance in the closed-loop could be reduced. Utilizing the previously introduced Monte Carlo simulation based optimization methodology new steady state operation points have been determined with multiplied economic performance with respect to the defined confidence level.

### 4.2. The Polymerization Process

 The process under consideration is a polymerization process controlled by a linear MPC, the previously mentioned DMC, [17]. The controlled system possesses all those difficulties which exist in an operating polymerization process.

#### 4.2.1. Process Description

The reactor which has been studied is a CSTR where a free radical polymerization reaction of methyl-metacrylate is considered using azobisisobutyronitrile (AIBN) as initiator, and toluene as solvent. The aim of the process is to produce different kinds of product grades. The number average molecular weight is used for qualifying the product and process state. The polymerization process can be described by the following model equations [18]:

(9) $$\begin{array}{c} \frac{dC_{m}}{dt}=-\left(k_{p}+k_{fm}\right)C_{m}P_{0}+\frac{F\left(C_{\min}-C_{m}\right)}{V}\\ \frac{dC_{I}}{dt}=-k_{I}C_{I}+\frac{F_{I}C_{\text{Iin}}-FC_{I}}{V}\\ \frac{dD_{0}}{dt}=\left(0.5k_{tc}+k_{td}\right)P_{0}^{2}+k_{fm}C_{m}P_{0}-\frac{FD_{0}}{V}\\ \frac{dD_{1}}{dt}=M_{m}\left(k_{p}+k_{fm}\right)C_{m}P_{0}-\frac{FD_{1}}{V}, \end{array}$$

where

(10) $$\begin{array}{c} P_{0}=\sqrt{\frac{2f^{*}C_{I}k_{I}}{k_{td}+k_{tc}}} \end{array}$$

The notation for the equations can be seen in Table 1. The number average molecular weight (NAMW) is defined by the ratio of *D*_1_/*D*_0_. By assuming an isotherm operation model the process model consists of four states, represented by four differential equation (9) [19]. For the integration, the MATLAB's built-in *ode45* function has been used, which is based on an explicit Runge-Kutta (4,5) formula. During simulations *T*_*s*_ = 0.03*h* is applied as sample time.

#### 4.2.2. MPC Controller Strategy and the Economic Performance Assessment

 The qualification of the product and process operation is based on the number average molecular weight. Thanks to the non-linear model equations the development economic performance turns into a highly non-linear optimization problem.

The control objective on the supervisory control level is to maximize the economic performance of the process. The objective function is formalized as

(11) $$\begin{array}{c} \underset{w_{\text{NAMW}}}{\max}E=P_{\text{onspec}}\cdot Q_{\text{onspec}}-P_{\text{offspec}}\cdot Q_{\text{offspec}}, \end{array}$$

where *P*_onspec_ (with the value of 10000) and *P*_offspec_ (with the value of 3500) are the prices of the polymer product which fulfill/not fulfill the product specifications; *w*_NAMW_ indicates the steady state setpoint. *Q* is the quantity of the polymer product, calculated with the following expression:

(12) $$\begin{array}{c} Q_{\text{polymer}}=F\cdot D_{1}. \end{array}$$

During the economic-oriented optimization, the following process constraints have to be considered:

(13) $$\begin{array}{c} 24000\leq \text{NAMW}\leq 26000. \end{array}$$

An important characteristic of the process is the increasing of product quantity when shifting the steady state operation closer to the lower limit, hence the optimal steady state operation point is expected near to the lower limit. The maximum probability of violating the process constraints is 1%, so the mentioned confidence level is 99%. The number of Monte Carlo simulations is 100, similarly to the previous case. The closed-loop variance of the process is caused by the noise added to the inlet monomer flowrate (*F*) with means of 0 and $\bar{\sigma }=0.014$. Other source of the closed-loop variance is the noise added to the controlled variable with the mean of 0 and $\bar{\sigma }=143$.

On the operative control level the previously proposed DMC is designed. The manipulated variable in the control strategy of the reactor is the initiator inlet flow rate. The tuning parameters of the applied DMC are *H*_*m*_ = 30, *H*_*p*_ = 3, and *H*_*c*_ = 3. The value of *λ* is chosen as 4 · 10^12^.

**Figure 10:**
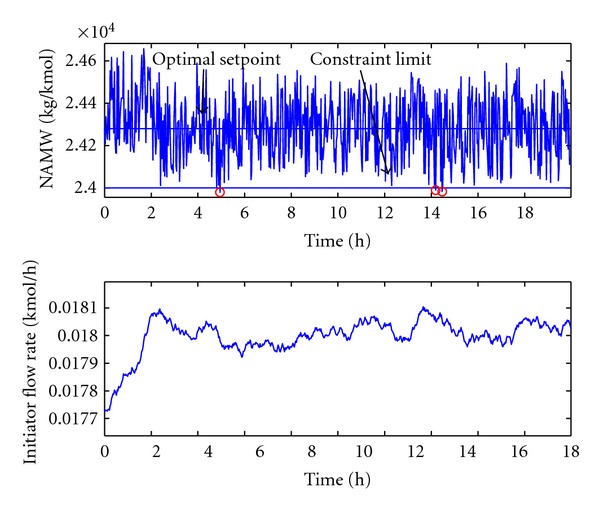
Improved case, optimal steady state operation point of PMMA reactor.

As base case the safest steady state operation point has been chosen which is in the middle of the specified operation range (*w*_NAMW_ = 25000). As the result of the economic performance optimization the optimal steady state setpoint is *w*_NAMW_ = 24300. Thanks to this setpoint modification the quantity of the produced polymer has been increased with 5% and throughout this the economic performance also increased with 5%. The result of the closed-loop simulation with the optimal setpoint is depicted in Figure 10.

The number of individual economic performance evaluations in the Monte Carlo simulation has been set to 100. In this case study there has been an attempt to apply quadratic programming as optimization algorithm. The same result has been obtained with quadratic programming but the computational demand was extremely high, almost 10 hour. By applying MADS, the computation demand has been decreased into 1 hour. As it can be seen, the quadratic programming might be applicable but its computation demand is exaggerated. The initial setpoint for the optimizer was set equal to the lower constraint of the output variable, *w*_0_ = 24000.

As Figure 10 shows that the frequency of constraint violation is conspicuously low, the process constraint has been violated only three times. It means 99.55% probability of not violating the limits, in contrast to the previously determined confidence level, which was 99%. It may happen since the off-specification product (products which do not fulfill the requirements) means extra outgoings in the economic objective function. Accordingly it is not worth to produce even just 1% off-specification product; however, this amount can be accepted technologically.

If the circumstances of the steady state operation change, there will be the need of redetermining the optimal operation point. This case is considered when the the deviation of the noise on the monomer inlet flow rate has been increased to $\bar{\sigma }=0.03$. Since the variation of the closed loop is increased, the optimal setpoint is determined further from the specification limit, *w*_NAMW_ = 24400. This way the economic performance is decreased compared to the previous case with 0.5%.

The results confirmed the assumption that the economically optimal operation is close to the process constraints. However, 99% was set as confidence level of limit violation; the way of formulating the economic cost function does not allow such a low quantity of off-specification product, since it causes extra outgoings during operation.

## 5. Conclusion

 In this paper an economic-oriented optimization framework has been introduced to determine optimal operation regimes of complex chemical process systems. Situations where the economically optimal steady state operation point is close to the technological limits of the operation have been studied. Due to process variance caused by unmeasured disturbances and measurement noise the determination of the optimal values of the controller setpoints is rather difficult since shifting the operation point closer to the process limits results in risk of violation of constraints related to process safety and product quality requirements. By formulating an economic objective function the performance and the risk level of the operation can be quantitatively evaluated. Monte Carlo simulation is applied with multiple runs of process model (augmented with the model of the control system) with economic performance assessment to handle the stochastic phenomena of process variance. Integrating the Monte Carlo simulation-based economic performance assessment tool into the Mesh Adaptive Direct Search (MADS) methodology can take process constraints and desired risk/confidence levels into consideration. Since MADS is the one of the recent gradient-free optimization methodology with high efficiency its application in Monte-Carlo simulation is much more effective than the classical gradient-based quadratic programming (SQP) by using MADS the time demand of optimization can be shortened to one-tenth of SQP.

The efficiency of the proposed framework is demonstrated throughout benchmark examples. In the first benchmark problem significant economic benefit was realized by finding the optimal setpoint signal after variance reduction. The process operation has been optimized at different confidence levels. Thanks to the efficiency of MADS the optimization has taken only 5 minutes; however, 100 iterations have been set in the Monte Carlo simulation. In case of the non-linear process the increase of economic throughput is not as significant as in the previous case, but with application of the proposed framework 5% profit increase can be obtained. The conditions of the optimization were similar to the previous case, 100 individual runs hve been set in the Monte Carlo simulation. Since the process model is non-linear the time consumption of the optimization is longer, almost one hour.

The application of the proposed methodology requires an existing process model with the description of the control system and detailed analysis of the process uncertainties. These modeling and analysis tools are widely available in advanced technologies thanks to the increasing interest for APC and Operator Training Systems (OTS). Another inevitable condition of application is the availability of an economic objective function. Although economic performance measures are frequently missing in current technologies, nowadays online economic performance monitoring is more and more indispensable. The aim of the application of these economic oriented monitoring tools is to avoid operations that are not economically or energetically optimal. Utilizing these performance assessment tools and integrating them with optimization the proposed framework is resulted.

## Figures and Tables

**Table 1:** Design parameters for MMA polymerization reactor.

*F*	1.0 m^3^/h
*C*_min⁡_	6.4678 kmol/m^3^
*C*_*I*in_	8 kmol/m^3^
*V*	0.1 m^3^
*M*_*m*_	100.12 kg/kmol
f*	0.58
*R*	8.314 kJ/kmol · K
*k*_*p*_	2.4952·10^6^ m^3^/kmol · h
*k*_*I*_	1.0224·10^−1^ 1/h
*k*_*fm*_	2.4522·10^3^ m^3^/kmol · h
*k*_*tc*_	1.3281·10^10^ m^3^/kmol · h
*k*_*td*_	1.0930·10^11^ m^3^/kmol · h
